# Early skeletal colonization of the coral holobiont by the microboring Ulvophyceae *Ostreobium* sp.

**DOI:** 10.1038/s41598-018-20196-5

**Published:** 2018-02-02

**Authors:** A. Massé, I. Domart-Coulon, S. Golubic, D. Duché, A. Tribollet

**Affiliations:** 1 Sorbonne Universités - Muséum National d’Histoire Naturelle, Laboratoire MCAM UMR7245 CNRS-MNHN, 63 rue Buffon, 75005 Paris, France; 2 IRD-Sorbonne Universités (UPMC Univ, Paris 06), Laboratoire LOCEAN UMR7159 CNRS-MNHN, 4 Place Jussieu, 75005 Paris, Cedex France; 3 Biological Science Center, Boston University, Boston, MA USA; 4 Aquarium Tropical, Palais de la Porte Dorée, 293 Avenue Daumesnil, 75012 Paris, France

## Abstract

*Ostreobium* sp. (Bryopsidales, Ulvophyceae) is a major microboring alga involved in tropical reef dissolution, with a proposed symbiotic lifestyle in living corals. However, its diversity and colonization dynamics in host’s early life stages remained unknown. Here, we mapped microborer distribution and abundance in skeletons of the branching coral *Pocillopora damicornis* from the onset of calcification in primary polyps (7 days) to budding juvenile colonies (1 and 3 months) growing on carbonate and non-carbonate substrates pre-colonized by natural biofilms, and compared them to adult colonies (in aquarium settings). Primary polyps were surprisingly already colonized by microboring filaments and their level of invasion depended on the nature of settlement substrate and the extent of its pre-colonization by microborers. Growth of early coral recruits was unaffected even when microborers were in close vicinity to the polyp tissue. In addition to morphotype observations, chloroplast-encoded *rbc*L gene sequence analyses revealed nine new *Ostreobium* clades (OTU99%) in *Pocillopora* coral. Recruits and adults shared one dominant *rbc*L clade, undetected in larvae, but also present in aquarium seawater, carbonate and non-carbonate settlement substrates, and in corals from reef settings. Our results show a substratum-dependent colonization by *Ostreobium* clades, and indicate horizontal transmission of *Ostreobium*-coral associations.

## Introduction

Reef-building scleractinian corals are associated with complex microbial communities, distributed in the mucus, tissue and skeletal compartments^1^. Microborer communities that actively penetrate carbonate skeletons by chemical means^2^ are among the relatively less studied partners of coral holobionts. Microborers (euendoliths) include filamentous cyanobacteria, fungi and algae (Chlorophytes and Rhodophytes)^2,3^. In living corals, the low-light environment inside the skeleton (often <1% of the photosynthetically active radiation)^4,5^ selects a few adapted euendolithic species. These microboring communities are usually dominated by the photosynthetic Ulvophyceae *Ostreobium* spp. (Chlorophyte)^6,7^, which was until recently called: *Ostreobium quekettii* Bornet et Flahault^8^. Dense populations of this euendolithic siphonous alga form visible green bands in the skeleton of adult colonies of massive, slow-growing corals of the genus *Porites* sp.^7,9^. In fast-growing branching corals such as *Stylophora pistillata*, euendolithic filaments gradually decrease in density upwards and the colored bands are absent^10^.

Molecular tools have during the past 10 years revealed a huge genetic diversity of Ulvophyceae that penetrate living scleractinian corals, with special attention to the siphonous euendolithic *Ostreobium*. The Ulvophyceae class contains multiple orders, including Ulvales (encompassing for instance families Ulvaceae, Phaeophilaceae), and Bryopsidales with 3 suborders (Ostreobidineae, Halimedineae and Bryopsidineae)^11^, each encompassing multiple families. Seven phylotypes of the RuBisCo large subunit of the chloroplast-encoded gene (*rbc*L) were recorded in *Ostreobium* colonizing 2 species of massive corals from the Red Sea along a depth gradient^12^. A recent study revealed four families within the Ostreobidineae suborder, using the *tuf*A plastid gene coding for the protein elongation factor EF-*Tu* as a metabarcode marker^13^ while exploring the diversity of *Ostreobium* spp. and associated endolithic green algae in limestone substrates. An environmental genome survey (plastid 16S rDNA and *rbc*L; nuclear18S rDNA)^14^ focused on *Ostreobium* phylogeny and ecology, and suggested a possible coevolution between *Ostreobium* and the main coral endosymbionts, the dinoflagellates *Symbiodinium* sp. In an independent study of the skeletal microbiome of massive slow-growing coral genera from various Pacific habitats, Marcelino and Verbruggen (2016)^3^ determined that the *tuf*A *Ostreobium* clade includes more than 80 taxonomic units at the near-species level. They combined *tuf*A with ribosomal RNA gene markers (nuclear 18S rDNA, and plastid 16S and 23S rDNA) and suggested that Ostreobidineae form a complex that has evolved over the last 500 million years. None of these studies, however, investigated the possible relationship between the developmental stages of the coral host and the *Ostreobium* clade diversity, and the colonization dynamics of microborers in corals remained unclear.

Early life stages of corals offer the unique opportunity to investigate the transmission of *Ostreobium*-coral associations and the mechanisms of holobiont assembly. The development of corals starts with a critical recruitment step, when a planktonic larva (planula) swimming in seawater settles to become the benthic primary polyp. Substrate settlement triggers larval metamorphosis and the onset of skeletal deposition, forming elements of the calcareous basal plate of the initial primary polyp within 24 h after settlement^15,16^. Secondary polyps then develop by clonal budding at the periphery of the primary polyp, forming the juvenile colony, which further grows into the adult colony, building the framework of reef ecosystems. In coral recruits, the polyp tissue layers cover tightly the growing carbonate skeleton, which may prevent colonization by microborers from the surrounding seawater. Thus, it is hypothesized that colonization occurs most likely through the substrate of larval settlement. However, this has not been experimentally tested. In reefs, coral larvae generally settle on biogenic carbonates such as dead coral rubble covered by coralline crustose algae^17^, substrates which are natural reservoirs for microborers^18,19^. However, coral larvae can sometimes settle on artificial, non-carbonate substrates^16^ covered by epilithic biofilm-forming microorganisms *a priori* free of microborers.

Here we aim to study the microborer colonization process of coral recruits and to address the following questions: (i) At what developmental stage does the colonization of coral skeleton by microborers take place, and how fast does it spread? (ii) What are the sources and reservoirs of colonizing microborers? (iii) Does the dominant *Ostreobium* clade depend on the site origin of the host and does it change in the course of the coral development?

We present a pilot study of colonization dynamics by microborers of early life stages of the coral *Pocillopora damicornis* type *beta*^20,21^. This species is a pioneer, fast-growing branching coral in tropical Indo-Pacific reefs^22^, with a life cycle that can be completed in captivity. Larval settlement experiments and long-term coral cultures in closed-circuit at ATPD-aquarium (Aquarium Tropical, Palais de la Porte Dorée, Paris, Fr) provide controlled conditions to study and map microborer abundance and distribution at unprecedented high temporal resolution in coral skeletons of three early life stages (7 days, one month, and three months post-metamorphosis), and in adult fully grown colonies. Two carbonate (dead *Porites* skeleton, calcite spar) vs two non-carbonate (plastic lumox^®^ -Sarstedt - and underwater paper) settlement substrates, previously pre-colonized (during 2–3 or 7 months) by microborers vs epilithic biofilms, were compared for their influence on microborer colonization of coral recruits. In addition to morphological criteria to detect *Ostreobium* filaments, the *rbc*L gene was amplified and Sanger sequenced to highlight the dominant *Ostreobium* clades in early coral life stages, their corresponding environmental seawater/settlement substrates, and adult colonies of *Pocillopora* corals from 3 French aquaria and 2 reef sites.

## Results

### Morphological microborer detection

**Figure 1:**
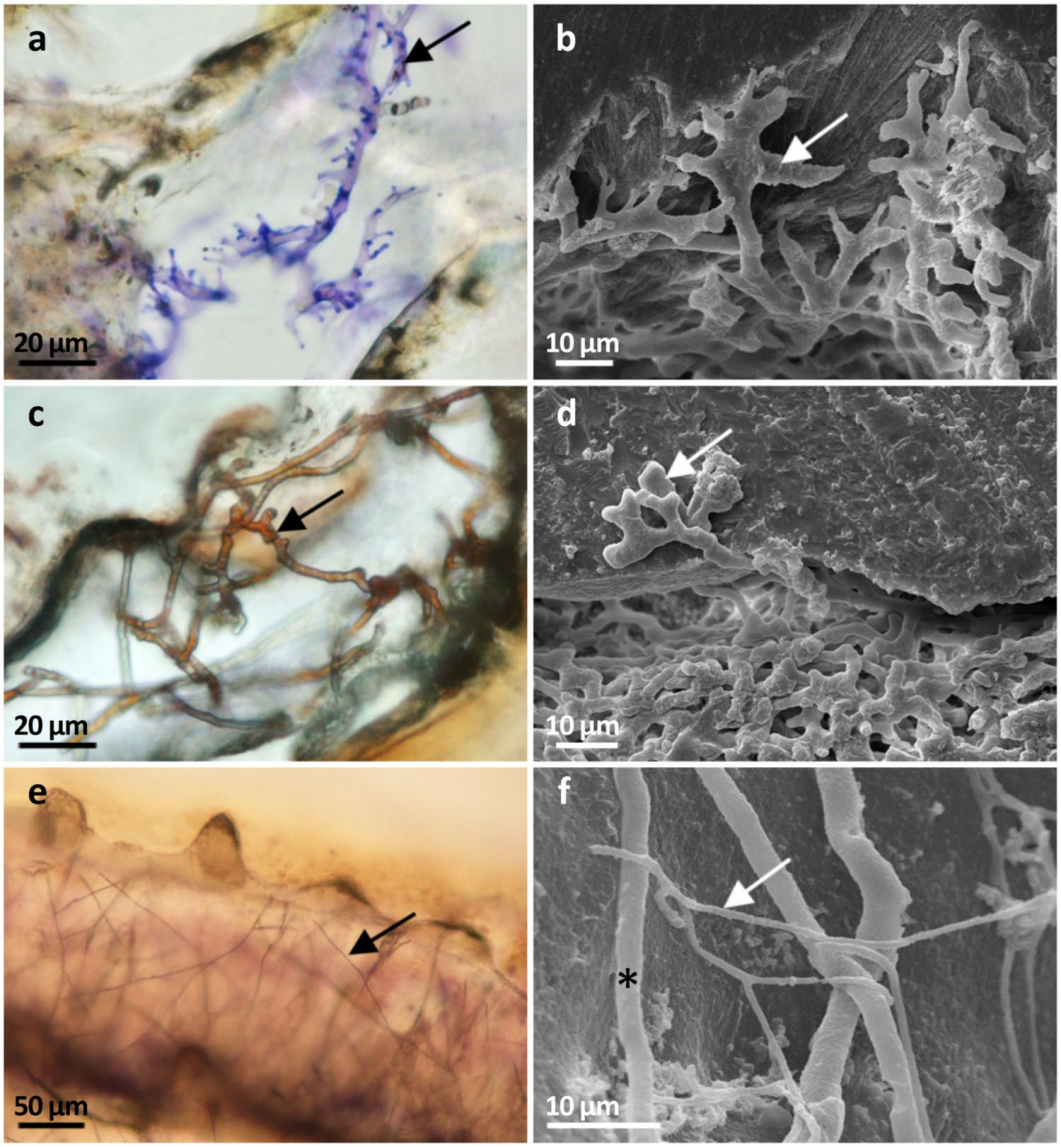
Filamentous microborers observed in coral skeletons. Light (**a, c, e**) and scanning electron micrographs of the associated microborings or galleries (**b, d, f**). (**a**) *Phaeophila* sp. in dead *Porites* substrate stained with toluidine blue (black arrow). (**b**) The corresponding resin- replicated galleries (white arrow). (**c**) *Ostreobium* sp. in 1 month old *P. damicornis* recruit stained with Grocott’s Methenamine Silver (black arrow). (**d**) The corresponding galleries in dead *Porites* substrate (white arrow). (**e**) Fungal hyphae in adult *P. damicornis* colony stained with toluidine blue (black arrow). (**f**) The corresponding galleries in dead *Porites* substrate (white arrow): fungi and *Ostreobium* galleries* are in close proximity.

In carbonate blocks of dead *Porites* skeleton, which were pre-exposed 2–3 and 7 months to colonization in the ATPD-Aquarium setting, the microboring Ulvophyceae *Phaeophila* and *Ostreobium* spp. and their associated undetermined fungi were regularly observed (Fig. 1). *Phaeophila sp*. filaments were recognized as branched with cells separated by cross-walls, each with extensions connected to the substrate surface (Fig. 1a, b). The siphonous *Ostreobium* sp. had characteristic polymorphic filaments with typical swellings and zig-zag branching pattern (Fig. 1c, d). Fungal filaments (~1 µm in diameter) showed perpendicular or dichotomous ramifications (Fig. 1e,f). In the calcite spar exposed 2–3 months, microboring communities included mostly the non Ostreobidinae *Phaeophila* and *Eugomontia* spp. After 7 months, these communities contained cyanobacteria (*Plectonema* sp. and *Hyella* sp.) but *Ostreobium* filaments were still not detected.

Epiliths had distinct morphologies and their assemblages varied with the type of experimental substrate. Epiliths on dead *Porites* skeletons pre-exposed 2–3 and 7 months mainly consisted of green algal turfs (Chlorophytes) with a few patches of crustose coralline algae (Rhodophytes). Calcite spar was mostly covered by crustose coralline algae, with a few Chlorophytes. Plastic and paper substrates pre-exposed for more than 6 months had surfaces colonized mostly by cyanobacteria (e.g. Oscillatoriales, *Spirulina*), diatoms, algal turfs, and fleshy or encrusting Chlorophytes and Rhodophytes (including crustose coralline algae). Interestingly, filaments typical of *Ostreobium* were detected within epilithic biofilms sampled at the surface of sand from aquariums at the ATPD site (A Couté, pers. com.).

**Figure 2:**
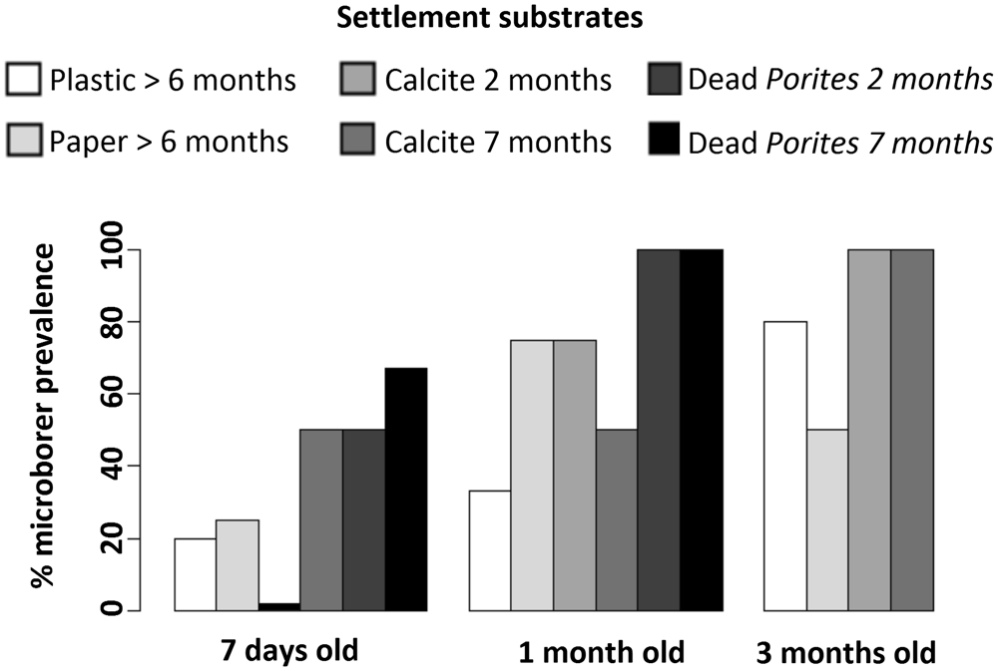
Substratum-dependent microborer colonization of coral recruits. Euendolithic filaments were detected by photonic and/or scanning electron microscopy in skeletons of *P. damicornis* (2 to 9 replicates per life stage and substrate type). One month old recruits developed on clean glass had 25% microborer prevalence. Data for 3 months old recruits on dead *Porites* are missing because full colonization was already reached on these substrates in 1 month old recruits (100% prevalence) and -given the limited availability of larval material- focus was given to the colonization process.

In the newly deposited skeletons of the settled coral recruits, microboring filaments observed were those of *Ostreobium* sp. accompanied by undetermined fungi. Note that filamentous structures were never observed in skeleton-free tissue of planktonic larvae. They were observed as early as the primary polyp stage in 7 days old recruits on both carbonate and non-carbonate (plastic/paper) substrates (Fig. 2). The prevalence of *Ostreobium* invasion increased with the age of recruits on carbonate substrates (dead *Porites* skeletons and calcite spars) to be fully colonized within 1 to 3 months. On non-carbonate substrates, microborer prevalence in recruits never reached 100% within the course of the experimentation (Fig. 2).

In colonies reaching adulthood, the microboring *Ostreobium* sp. and undetermined fungi were always observed in abundance (100% prevalence), both in the growing branch tips (apexes) within the tissue-covered skeleton and in basal skeleton bare of coelenterate tissue. *Phaeophila* sp. was very rarely observed and only in the coral bare bases.

### Dominant *rbc*L clades of *Ostreobium*

Cloning an almost full length (~1380 bp) fragment of the *Ostreobium rbc*L gene yielded 113 sequences distributed among 12 clades, with >99% sequence similarity intra-OTUs. Table 1 presents the time-dependent succession of *Ostreobium* clades in the developing recruits and adult corals, as compared to larval settlement substrates and seawater. Supplementary Fig. 1 illustrates the *rbc*L Maximum-Likelihood phylogeny of *Ostreobium* in our samples (1a) and the clade distribution mapped according to environmental sources (1b) and site origins of *Pocillopora* hosts (1c).

**Table 1:** Coral samples and environmental controls used for *rbc*L detection of dominant *Ostreobium* clades.

	Sampling site	Sample type (n replicates)	Positive *rbc*L amplification	Cloned *Ostreobium*	*Ostreobium* OTUs (>99%)	Cloned non-Ostreobidinae Ulvales and Bryopsidales
**Experiment**	ATPD aquarium	Planula larvae (n = 2 x2*)	0/2	/	/	/
		Early metamorphosis (n = 2 x3*)	0/2	/	/	/
		2 days primary polyp (n = 2 x4*)	0/2	/	/	/
		10 days old recruits growing on plastic (n = 3 x3*)	2/3	7/9	P1, P10, P11	2/9
		1 month old recruits growing on paper (n = 2)	2/2	4/11	P1, P6	7/11
		1 month old recruits growing on plastic (n = 1 x3*)	1/1	0/6	not detected	6/6
		3 months old recruits growing on plastic (n = 1)	1/1	1/5	P8	4/5
		2 months old recruits growing on dead *Porites* (n = 1)	1/1	6/7	P1, K	1/7
		Living adult branches of *Pocillopora damicornis* (n = 5)	5/5	27/27	P1, P2, P3	0/27
		Plastic (>6 months) (n = 3)	1/3	2/11	P1, P8	9/11
		Paper (>6 months) (n = 2)	1/2	4/11	P10	7/11
		Dead *Porites* (2 months) (n = 1)	1/1	9/11	P1,P2,P5	2/11
		Dead *Porites* (8 months) (n = 1)	1/1	5/7	P1	2/7
		Seawater (1L filtered on 0.2µm membrane) (n = 3)	1/3	6/6	P1, P5, P9	0/6
**Controls**	Océanopolis aquarium	Living adult branches of *Pocillopora damicornis* (n = 3)	3/3	14/14	P1, P3	0/14
		Seawater (3.5L filtered on 0.2µm membrane) (n = 1)	1/1	6/6	P1	0/6
	Canet aquarium	Living adult branches of *Pocillopora damicornis* (n = 3)	3/3	15/15	P2, P4	0/15
	IUI Reef, Eilat (Israël)	Living adult branches of *Pocillopora verrucosa* (n = 10)	2/10	6/8	P1	2/8
		Seawater (3L or 2.5L filtered on 0.2µm membrane) (n = 2)	0/2	/	/	/
	New-Caledonian Reef	Living adult branches of *Pocillopora* sp. (n = 6)	1/6	1/NA	P7	NA

(*) early coral life stages were pooled by 2–4 individuals to increase biomass for DNA extraction. NA = non-available data.

In settlement substrates, dead *Porites* skeletons pre-exposed 2 months contained three *Ostreobium* clades: P1 (56%), P2 (22%) and P5 (22%). These clades are closely related to *Ostreobium rbc*L clades K (KT280005), E (KT280002), and D (KT280001) identified in massive *Porites* sp. corals from the Red Sea, with 97%, 90% and 97% similarities, respectively. In the dead *Porites* skeleton that was exposed more than 8 months, only clade P1 was detected. In the calcite spar pre-exposed to colonization for 4 and 10 months, *Ostreobium* was not detected.

In the epilithic biofilms that formed on the surface of the settlement substrates, three *Ostreobium* clades were detected: P1 and P8 on plastic, and P10 on paper. Clade P8 and P10 are 98% similar and both clades are closely related to *rbc*L sequences of *Ostreobium quekettii* strains (96% similarity, FJ715720, FJ535853). The *rbc*L primer set also amplified other non-Ostreobidinae Ulvophyceae (i.e. Ulvales, Bryopsidales other than *Ostreobium*). Moreover, epilithic Rhodophytes such as Gigartinales and Hapalidiales (encompassing a few crustose coralline algae species) were detected on non-carbonate substrates with specific primers (SPF30 and SPR40^23^, data not shown).

Interestingly, *Ostreobium* clades P1 (66%) and P5 (17%) were also detected in the environmental seawater of ATPD-Aquarium, but seawater contained an additional clade, P9 (17% - with 97 and 96% similarities to *rbc*L sequences of *Ostreobium* clade P5 and D, respectively). Clade P1 was also detected (100%) in seawater of the Océanopolis-Aquarium (Brest; Supplementary Fig. 1b).

In coral recruits, *Ostreobium* clades varied with the host life stage and the settlement substrate (Table 1). In swimming planktonic larvae (which lack skeleton), and early settling larvae (1–2 days into metamorphosis, with only partially formed skeletal basal plate), the *rbc*L gene was not amplified. In 10 days old recruits settled on plastic, *Ostreobium* clades P1 (43%), P10 (43%) and P11 (14%) were detected. Clade P10 and P11 are closely related to each other (98.6% similarity). Note that these clades were also detected on non-carbonate substrates (see above). In 1 month old recruits settled on paper, two *Ostreobium* clades were detected, including P1 (75%, also recorded at 10 days, in dead *Porites* substrates and in seawater), and a new clade, P6 (25%) related to clade D from living *Porites* coral in the Red Sea (95% similarity). In 1 month old recruits on plastic, *Ostreobium rbc*L sequences were not detected. However, *Ostreobium* was again detected in 3 months old recruits growing on plastic, which contained clade P8 found on plastic substrate. Finally, in 2 months old recruits on dead *Porites* substrate pre-exposed 2 months to colonization, *Ostreobium* clades P1 and K were detected (97% similarity) (Supplementary Fig. 1). Note that most *Ostreobium* clades were shared by recruits and both carbonate and non-carbonate settlement substrates.

In adult *Pocillopora damicornis* from long-term aquarium cultures, *Ostreobium* was detected in all apexes of colony branches, mostly represented by a single clade, and sometimes two (Table 1). Recovered clade diversity varied with site location (Supplementary Fig. 1c). Clade P1 found in coral recruits, seawater, plastic and dead *Porites* substrates, also occurred frequently in adult colonies from ATPD (70%). In contrast, it was rare in colonies from Océanopolis (7%) and absent in colonies from Canet aquarium. In Océanopolis colonies, clade P3 - distantly related (96.7% similarity) to *rbc*L clade F from Red Sea *Porites* sp. (KT280003) - was the most frequent (93%), whereas it was less frequent at ATPD colonies (26%) and absent from Canet aquarium. In colonies from Canet, clade P4 - related (94% similarity) to *rbc*L clade A from Red Sea *Porites* sp. (KT279998) - had the highest occurrence (93%). Clade P2 was recorded at low-frequencies in adult colonies from both Canet (7%) and ATPD (4%), and was also detected once in dead *Porites* substrate pre-exposed 3 months at the ATPD. In addition to the *rbc*L marker, the *tuf*A gene was amplified in an adult *P. damicornis* from ATPD aquarium (with primers Oq-tuf^3^ and *tuf*AR^24^). The obtained *tuf*A sequence (~420 bp) was 87% similar to *Ostreobium tuf*A in the Odoceae family^13^.

To compare the dominant *Ostreobium* associates of *Pocillopora* corals from the *ex-situ* aquarium settings with those in natural reefs (Supplementary Fig. 1c), the *rbc*L gene was amplified in 2 out of 10 adult colonies of *Pocillopora verrucosa* collected at the IUI reef in Eilat, Red Sea. Molecular analyses revealed the presence of only P1 clade. A distinct, *rbc*L clade P7 was detected in *Pocillopora* sp. from a New-Caledonian reef site (1 out of 6 colonies), 94% similar to clade P3 detected in ATPD and Océanopolis colonies (Supplementary Fig. 1a,c).

### Abundance and heterogeneous distribution of microborers

**Figure 3:**
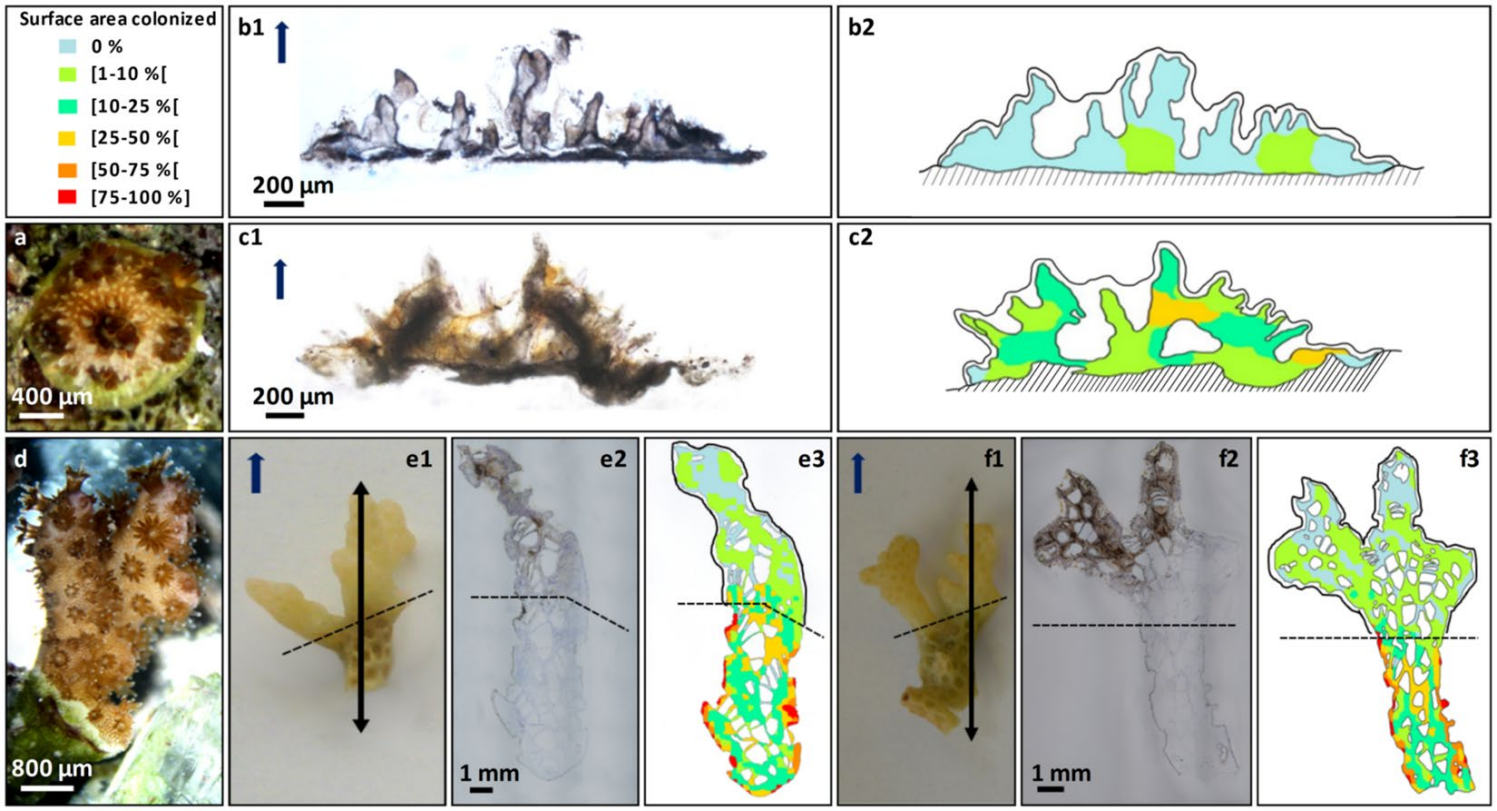
Colonization dynamics of skeletons of coral recruits and adult colonies by microborers. (**a**) One month old *P. damicornis* recruit. (b1) Thin section and (b2) corresponding distribution/abundance mapping of microborers in one month old recruit settled on non-carbonate (plastic) or (**c1**,**c2**) carbonate substrate (dead *Porites* skeleton). (**d**, e1, f1) Adult *P. damicornis* colony branch. (e2) Thin section and (e3) corresponding distribution/abundance mapping of microborers in an adult colony; (f2, f3) also illustrated for another adult sample. The higher is the abundance, the warmer is the color code on maps. Dotted line represents the boundary between tissue-covered skeleton (apex) and dead skeletal base. Orientation of thin sections is parallel to coral vertical growth axis (dark blue arrow).

Microborer filaments were more abundant in recruits growing on porous carbonate substrates (dead *Porites* skeleton) than on other substrates (Fig. 3). This variability was due to the level of pre-colonization by microborers in settlement substrates, and the stage of development of coral recruits (Fig. 3b1,b2 and c1,c2). Note that, the measured Relative Surface of Microborer colonization (RSM) in recruits growing on non-carbonate substrates was low and stable over time (0.44 ± 0.05%, n = 1365 measurements), while colonization increased and varied greatly with time in juveniles growing on carbonates. In 1 month old recruits on dead *Porites* skeletons, RSM increased from 4.33 ± 0.49% after 3 months of exposure (n = 191 measurements) to 10.95 ± 1.81% after 7 months (n = 66 measurements). In 3 months old recruits on calcite spar, RSM increased slowly, from 1.04 ± 0.22% after 3 months (n = 185 measurements) to 2.83 ± 0.38% after 7 months (n = 347 measurements). In adult colonies, colonization (Fig. 3e1,e3 and f1,f3) was significantly higher in the basal areas of the coral skeleton, with a minimum of 8.75 ± 0.61% (n = 131 measurements) and maximum of 30.87 ± 1.76% (n = 179 measurements), than at the branch apexes where the minimum was 2.04 ± 0.23% (n = 120 measurements) and the maximum 4.76 ± 0.48% (n = 399 measurements). Generally, despite spatial heterogeneity, colonization of corals was highest in dead bases compared to living apexes of adult branches, and compared to juveniles.

**Figure 4:**
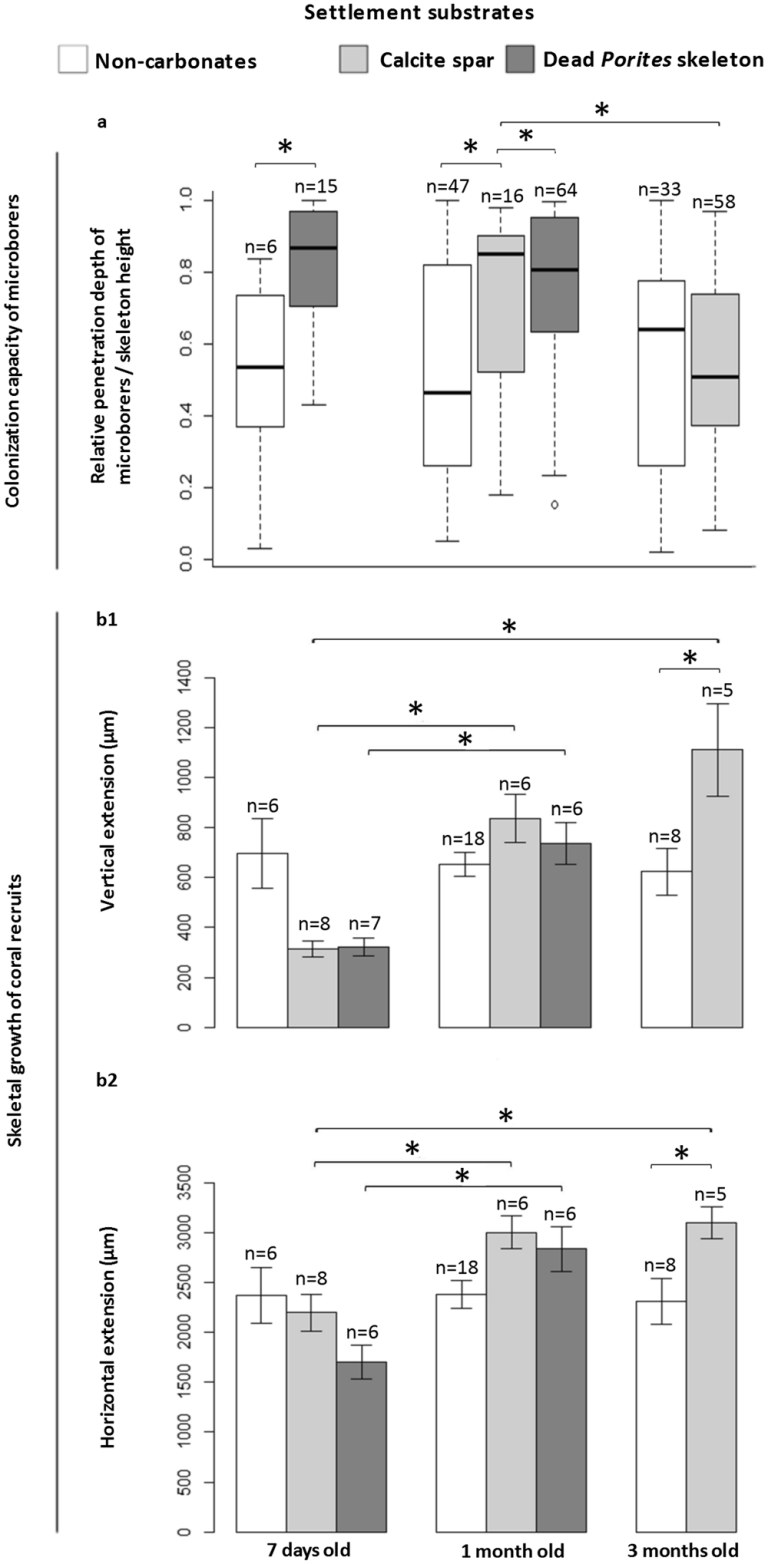
Microborer penetration into rapidly extending skeletons of early coral recruits. (**a**) The colonization capacity is reported as relative depth of penetration of microborers to skeletal height (relative DP) and varies among larval settlement substrates; n = number of measurements of depths of penetration. (**b1**) Corresponding vertical and (**b2**) horizontal extension rates of n replicate coral recruits, with standard error. *Significant differences (P < 0.05). Data for 3 months old recruits on dead *Porites* are missing because monitoring of larval development on these substrates was stopped at 1 month when prevalence of microborers in coral skeleton reached 100% (see Fig. 2).

Similar to microborer abundance, the depth of penetration of filaments inside skeletons of recruits varied significantly depending on the type of settlement substrate and the extent of its pre-colonization (Fig. 4). In dead *Porites* skeletons, microborer abundance and penetration were much higher than in calcite spar (depth penetration of 460 ± 49 vs 71 ± 11 µm - n = 12 measurements- after 3 months of exposure, and 553 ± 41 vs 128 ± 10 µm - n = 12 measurements- after 7 months respectively). In coral recruits, the DP ratio (depth of microborers penetration/height of coral skeleton along the vertical axis) ranged between 0.02 (initiation of colonization) and 1 (microborers very close to the tissue). Penetration was maximum (DP ratio of 0.79 ± 0.05- n = 15 measurements) at the primary polyp stage, for recruits on dead *Porites* skeletons. In contrast, penetration was lowest and stable over time (DP ratio of 0.53 ± 0.03- n = 86 measurements) for recruits on non-carbonate substrates. In adults, despite a highly heterogeneous microborer distribution throughout the skeleton, thin filaments (most probably fungi based on their morphology) were also observed colonizing all the way up the skeletal septae and spines, just underneath coral tissues (Fig. 3f3 and e3).

After grouping vertical and horizontal coral extension data for each type of settlement substrate and for the same stage of colony development (as no significant differences were found, Mann and Whitney or Kruskal-Wallis tests, P > 0.05), our results show that juvenile sizes were stable on non-carbonate substrates between 7 days and 3 months (Fig. 4b1,b2). In contrast, juveniles settled on carbonates extended significantly, both vertically and horizontally, especially during the first month, with a faster vertical than horizontal extension. Indeed, while horizontal extension increased only by a factor 1.4 to 1.7 for recruits on calcite spar and dead *Porites* skeleton, respectively, the coral vertical extension was multiplied by a factor 2.7 and 2.3 respectively.

## Discussion

At the reef scale, *Ostreobium* sp. is the main agent of microbioerosion^25,26^, i.e. the principal carrier of biogenic dissolution of carbonates. Despite host skeletal erosion, a few authors have proposed that this phototrophic euendolith may improve the survival of thermally stressed corals during bleaching events (e.g. *Oculina patagonica* in the eastern Mediterranean Sea) via a transfer of alternative photoassimilates to the host^27,28^. To clarify their role in the coral holobiont, it is important to understand the sequence of microborer colonization of the skeletons of living corals. Evaluating the timing of the establishment of coral-microborer association, the potential colonization of specific *Ostreobium* clades during that process, and its effect on early coral life stages is especially relevant in the current context of anthropogenic global changes. Carbonate dissolution of reef substrates by microborers is indeed enhanced by eutrophication and rising aqueous pCO_2_^29,30^ so that the combination of several environmental factors including rising sea surface temperatures threaten the coral recruitment and growth^31,32^.

Here, we provide the first evidence on the entry of the euendolithic alga *Ostreobium* during the early ontogeny of a coral host. We show that colonization occurs within a few days (between 2 and 7 days) following the larval settlement on pre-colonized substrates, at the onset of skeletal deposition and basal plate formation in the primary polyp. Microboring filaments penetrate from the settlement substrate into the skeletons of coral recruit in a similar way as previously observed in live thalli of the coralline alga *Hydrolithon onkodes*^18^.

The observed phototrophic (and phototropic) euendolith, *Ostreobium* must keep up with the vertical extension of its also largely phototrophic host to survive, taking in consideration that coral’s tissue-shaded skeletons constitute an extreme low light environment^4,5^. This adaptation was first suggested by Le Campion-Alsumard *et al*.^7^ while explaining the pattern of green bands in adult colonies of the massive coral *Porites*, which correlated with periods of slower coral growth, permitting dense growth of multiply ramified *Ostreobium* filaments. Here, in fast accreting adult colonies of the branched *Pocillopora damicornis*, no colored bands underneath coral tissues were observed, instead, the euendolith abundance decreased by a factor 6 towards the tissue-covered branch tips. This pattern is similar to that found in the branching species *Stylophora pistillata*^10^, where fast growth leaves the euendoliths behind, resulting in an upward-progressive filament density decrease. However, no such virtual filament ‘dilution’ effect could be observed during the early deposition and expansion of carbonate in juvenile coral colonies. The rapidly growing coral recruits developing on heavily pre-colonized dead *Porites* were invaded by euendolithic microorganisms, which expanded through the polyp skeletal basal plate up to the areas in close vicinity to the coral tissue.

We show for the first time that this early colonization did not slow the host extension rates, indicating that the fitness of recruits was not altered by early assembly of the coral-microborer association. Pre-colonized dead *Porites* skeleton fragments with highest concentration of endolithic *Ostreobium* proved also to be the best source for euendolith recruitment to coral juveniles that settled on them, providing a faster and more extensive microborer colonization compared to calcite and non-carbonate substrates. This pattern, together with similar succession of microboring communities in dead *Porites* skeletons, becoming mature i.e. dominated by *Ostreobium* after 7 months of exposure in our controlled aquarium settings (ATPD), just as shown in natural coral reefs^26^, indicates the representativeness of our *ex-situ* model to *in situ* processes. A really unexpected result of the present study was the early colonization by microborers of coral juveniles settled on non-carbonate substrates (plastic and paper). We checked that the soft tissues of these recruits covered the entire skeleton and were not damaged. We then hypothesized that some microborers were present on non-carbonate substrates as epilithic forms. Indeed, Golubic *et al*.^33^ showed that *Ostreobium* filaments can exit coral pores and become temporary crypto-endolithic organisms. Kobluk and Risk^34^ also suggested that *Ostreobium* filaments can exit their carbonate substrate to become epilithic. We prove the correctness of this hypothesis by observing filaments typical of *Ostreobium* within epilithic biofilm developed at ATPD, and by amplifying the *Ostreobium rbc*L gene on non-carbonate substrates covered by natural biofilms (plastic and paper) and in seawater. *Ostreobium* filaments have been reported to display sporangial bags, allowing the production of quadriflagellate zoospores in the environment^35^. However, the life cycle of *Ostreobium* remains poorly known, especially regarding the way spores are expelled from the presumed sporangial bags located inside the substrate into the seawater. We propose that spores or detached fragments of this siphonous alga became trapped in the epilithic biofilms and started to develop into filaments while inside the primary corallite of the coral recruits (skeleton of the primary polyps).

The molecular aspects of our study confirm that microboring communities in living corals are reservoirs of new *Ostreobium* diversity. Indeed, 9 new *Ostreobium rbc*L clades (OTU99%) were detected in branching *Pocillopora damicornis* (type *beta*^21^) from long-term aquarium cultures, *P. verrucosa* from Eilat reef (Haplotype A and E^36^) and *Pocillopora* sp. from a New Caledonian reef, of which only clade K (OTU99%) was previously known from a shallow water massive *Porites* coral from the Red Sea. These results provide new information on the diversification of the genus *Ostreobium*, adding to the growing datasets of operational taxonomical units, which have recently been reported from the skeleton of mostly massive scleractinian corals, using the *rbc*L gene as well as complementary gene markers^3,11,13,14^. Here, the association of *Pocillopora* corals with *Ostreobium* clades seems quite variable and substratum-dependent. It should be noted that the *Ostreobium* clades present in settlement substrates may be affected by the geographic distribution^14^ of these clades and their depth distribution^12^. Further investigations will require further broad scale sampling using for example metabarcoding. The strongly supported *Ostreobium rbc*L clade P1 (OTU99%, close to clade K) dominated coral grown in ATPD aquarium, both in adults and juveniles, and was absent from larval stages but present in environmental reservoirs such as dead *Porites* carbonate substrates as well as non-carbonate substrates and seawater. Altogether these data support horizontal intergenerational transmission of the *Ostreobium*-coral association. Besides, the P1 clade was confirmed in adults from another aquarium settings (Océanopolis) and *in situ* in Red Sea shallow reef settings in a parent *Pocillopora verrucosa* species, suggesting widespread occurrence in *Pocillopora*. Future studies are needed to further investigate this molecular diversity and the multiple factors and communication mechanisms potentially driving the selection of *Ostreobium* clades at various stages of the host development. More generally, the functional interactions between microborers of specific *Ostreobium* clades, and their living coral host need to be studied to understand the capacity of corals to adapt to changing environmental conditions.

In conclusion, this study provides novel information on the timing of microborer colonization of early coral recruits, which occurs as early as 7 days post-metamorphosis in the primary polyp, before budding into a juvenile colony. Recruits settled on non-carbonate substrates are also colonized by *Ostreobium* clades, indicating the existence of life stages (propagules) in seawater and among epilithic biofilms which are able to penetrate newly deposited carbonate skeletons. Occurrence analyses indicate substratum-dependent *Ostreobium*-coral associations, with a widespread *rbc*L clade P1 of *Ostreobium*, detected both in environmental reservoirs and across *Pocillopora* sp. benthic life stages. Its dominance in aquarium microcosms suggests that it may be an ecologically dominant strain in *Pocillopora* corals but this needs further exploration in natural reef settings. Combined together this data show horizontal transmission of the *Ostreobium*-coral associations. Interestingly, the presence and abundance of microborers did not affect the coral skeletal extension rates, and deeply penetrating borings were observed in close proximity to coral tissues. These findings reveal the early incorporation of microborers into the coral holobiont, and have implications for a potential role of these microbial associates on coral host health and development.

## Materials and Methods

### Biological material and experimental design

Planula larvae were collected from adult colonies of the coral *Pocillopora damicornis* type *beta*^21^ propagated in long-term cultures in the ATPD-Aquarium in Paris and in Océanopolis-Aquarium in Brest (France). The planulae emitted few days before the full moon were put in contact with experimental settlement substrates to induce metamorphosis and recruitment in 400 ml seawater in glass beakers maintained in 2 small tanks of 30 L (with gentle air bubbling and seawater half-renewed daily). These tanks were connected to a 750 L tank containing adult colonies and settlement substrates pre-exposed during 2–3 months or 7 months to colonization by epilithic biofilms and the microborers growing in the tank. Two carbonate substrates were tested, a non-porous calcite spar (Corps & Ames, Belgium) and blocks (2 × 2 × 2 cm^3^) of naturally porous ‘dead coral’ skeleton from *Porites* sp., bleached to remove organic matter and potential algal propagules. Two non-carbonate porous substrates were also tested, plastic 5 cm diameter lumox^®^ Petri dishes (Sarstedt, France) and underwater paper. Non-porous clean glass, free of microborers or epiliths, was additionally tested for larvae which settled on submersed beaker (sampled at 1 month old).

All studied samples were exposed to the same constant seawater conditions during the experiment (pH 8.2 ± 0.2, temperature 25 ± 0.1 °C, salinity 35, calcium 400–450 mg/L, magnesium 1300 ± 60 mg/L, nitrate 1 ± 0.7 mg/L and phosphate 0.005 ± 0.008 mg/L). Light intensity measured with an immerged spherical LI-COR quantum-meter was in the range 35–60 µmol photons.m^−2^.s^−1^ in the beakers containing larvae and 120–140 µmol photons.m^−2^.s^−1^ in the larger tank near the top surface of adult coral colonies. Pre-colonized settlement substrates were exposed to light intensities varying between 60–90 µmol photons.m^−2^.s^−1^ (in the larger tank) while non-carbonate substrates were exposed to similar irradiance as the developing coral recruits (inside beakers).

Upon contact with the experimental substrates, the larvae metamorphosed into primary polyps (within ~2–3 days of contact). Samples for microborers and *Ostreobium* study were collected from four stages of coral development: 7 days, 1 and 3 months old and adult branches (with living tissue-covered apex, and dead base without coral tissues), as well as from the settlement substrates and environmental seawater. For morphological analyses of microborers, samples were fixed in 2.5% glutaraldehyde, 1% paraformaldehyde, in 0.6 M sucrose −0.1 M Sörensen phosphate buffer (pH 8). For molecular detection of *Ostreobium*, samples of corals, settlement substrates and seawater (1–3 L) filtered on 0.2 µm membranes, were frozen at −20 °C or put in ethanol 95%.

As a control regarding *Ostreobium* clades (*rbc*L) detected at ATPD-Aquarium (n = 5), we also analyzed adult colony replicates from the same *beta* lineage of *Pocillopora damicornis* host, propagated in long-term cultures in two other French aquaria, i.e. at Océanopolis Brest (n = 3) and Canet-en-Roussillon (n = 3), with seawater intake from the North-East Atlantic (Rade de Brest) and the Western Mediterranean Sea (Golf du Lyon), respectively. ATPD-aquarium colonies from *Pocillopora damicornis* type *beta* coral are propagated via fragmentation and natural reproduction from initial colonies originating from Indonesia (imported under CITES permit FR01081 00211/12-i and exchanged between Océanopolis and ATPD aquaria). Additionally, reef samples were analyzed for comparison with aquarium-grown corals. They included *P. verrucosa* samples (haplotypes A and E^36^) collected at 6–10 m depth at Eilat in the Red Sea (n = 10 at InterUniversity Institute for Marine Sciences reef (IUI); #2011/38182 collection permit number) and *Pocillopora* sp. samples from the 12 m depth reef of Ugo in New Caledonia (n = 6; #3042–2012/ARR/DENV collection permit number from Province Sud, NC). Coral host lineages were checked by sequence analysis of the amplified mtORF mitochondrial taxonomical marker^21,36^.

### Morphological microborer detection

Early coral recruits (2 to 9 replicates per life stage and substrate type, total n = 65), their carbonate settlement substrates (total n = 15), and branches of adult colonies (n = 3) were prepared for thin sections to observe microborers in light microscopy (Nikon Eclipse LV100) and their traces in scanning electron microscopy (SEM, ZEISS Evo.LS.15; Alysés platform, IRD Bondy, France). Preserved samples were dehydrated in ethanol (50%, 75%, 100%), and vaccum-embedded in Struers^®^ epoxy resin at room temperature. Thin sections were then cut along the coral vertical growth axis using a circular diamond saw (STRUERS), and polished down to 20–30 µm on a BUEHLER^®^ polisher or by hand. Sections were slightly etched with 10% hydrochloric acid during a few seconds, rinsed with MilliQ water, stained with 5% toluidine blue or Grocott’s Methenamine Silver, and then coverslipped in Araldite^®^. Alternately, some unpolished thin sections were bleached with sodium hypochlorite, acid-etched, and gold-coated on SEM-stubs. Microborer colonization of recruit skeletons was monitored until prevalence reached 100%, i.e. the level equivalent to those in fully grown colonies.

Biofilms developed on non-carbonate substrates (paper, plastic) were also observed fresh or after aldehyde fixation with an Olympus inverted microscope, to survey the dominant epilithic morphotypes (e.g. cyanobacteria, turf green algae, encrusting Rhodophytes) and to detect potential forms of *Ostreobium*.

Distribution and abundance maps of microborer filaments in coral skeletons were created according to Godinot *et al*.^10^. Six ranks of abundance were used (0%, [1–10%[, [10–25%[, [25–50%[, [50–75%[, [75–100%]) and represented the Relative Surface area of coral skeleton colonized by Microborers (RSM). Vertical and horizontal extensions of juveniles were determined on thin sections (with ImageJ software, N.I.H., USA) allowing the calculation of the ratio DP, i.e. ‘penetration depth of microborers/height of coral skeleton (in the vertical axis)’. A relative DP of ~1 indicated a complete penetration of microboring filaments from the settlement substrate up to coral tissue vicinity while a relative DP of ~0 indicated a limited penetration of filaments into coral skeletons.

### Molecular detection of the microboring *Ostreobium*

Coral tissues covering apexes of adult samples (n = 27) were removed with a WaterPik^®^^37^ using a pressurized jet of Phosphate Buffered Saline (10 mM pH 7.4) to limit contamination of skeleton by the tissue and its dinoflagellates. Similarly, epilithic biofilms on carbonate substrates, i.e. dead *Porites* skeleton (n = 2) and calcite spar (n = 2), were removed by gentle scraping with a scalpel before applying the WaterPik^®^, to limit contamination of endoliths by epilithic organisms. Skeletons of adult colonies and carbonates substrates (wet weights ~606 ± 178 mg and ~1.2 ± 0.09 g, respectively) were then ground down to a fine powder in autoclaved mortar and pestle cooled by liquid nitrogen. Coral recruits (n = 8, wet weight ~15 ± 2.3 mg) were sampled with a scalpel under the stereomicroscope (avoiding contamination from substrate of fixation), and crushed in Eppendorf with autoclaved piston pellet. Replicates of the planula larvae (n = 2), early metamorphosis stages (n = 2), or 2 days primary polyp (n = 2), containing tissue with none or very few skeletal granules^16^, were pooled by 2–4 individuals to increase the biomass available for *Ostreobium* detection (wet weight ~1 mg/planula). Membrane filters (0.2 µm) of environmental seawater (total n = 6), and pieces of paper or plastic, i.e. non-carbonate substrates (total n = 5), were cut into small pieces before DNA extraction. Each sample category (planula larvae; recruits; environmental seawater; settlement substrates; adults from each aquarium or reef site) was extracted in separate experiments, to avoid potential cross-contamination.

Total DNA of each sample was extracted using PowerSoil^TM^ DNA Isolation Kit (Mobio Laboratories Inc., CA). For planula larvae to juvenile stages, with very low biomass, glycogen was added as a nucleic acid carrier (30 µg/ml final concentration) before DNA precipitation. A ~1380 nt almost full length fragment of the chloroplast-encoded *Ostreobium rbc*L gene (1428 nt^14^) was amplified with the following oligonucleotide primer pair: *rbc*L7F [5′CCAMAAACWGAAACWAAAGC 3′]^38^ and *rbc*L1391R [5′TCTTTCCAAACTTCACAAGC 3′]^11^ specific to the Bryopsidales order within the Ulvophyceae. Amplification reactions were performed in 25 µl volume containing 1 µl DNA extract template, 0.5 µl of each primer (10 µM final concentration), 2 µl MgCl_2_ (25 mM), 0.5 µl dNTP (10 mM), 5 µl of 5X GoTaq  Flexi Buffer, 0.125 µl enzyme GoTaq^®^ G2 Flexi DNA Polymerase (Promega, France) in sterile water. Cycling conditions were 4 min at 94 °C, 40 cycles of [30 s at 94 °C, 45 s at 55 °C, 90 s at 72 °C], and 5 min terminal extension at 72 °C. Amplified fragments were visualized in 1% agarose gels with SYBRGold, and purified (NucleoSpin^®^ gel and PCR clean-up kit, Macherey-Nagel, France). Reducing the number of cycles did not yield enough DNA for visualization. To increase the recovered diversity, amplicons were pooled from 2 to 6 independent positive PCR reactions, before cloning into pGEM-T easy vector plasmids and competent *Escherichia coli* JM109 cells (Invitrogen, France). DNA plasmids of insert-containing colonies were extracted using Wizard Plus SV Minipreps (Promega, France) and 4 to 11 clones per amplicon were Sanger-sequenced in one or both directions at Eurofins Genomics (Germany). The cloned sequences were checked manually then assembled and aligned with their closest matching *rbc*L sequences retrieved by BLASTn in GenBank database. A 578 nt alignment of our 113 cloned *Ostreobium* sp. *rbc*L sequences was generated, to allow comparison with reference sequences from type strains or clones from Red Sea massive corals, using ClustalW Multiple Alignment tool in MEGAv6 software. Phylogenetic reconstruction was carried out using the maximum likelihood algorithm, with Kimura 2-parameter distance^39^ and 500 bootstraps. Sequences of *rbc*L from the Bryopsidale *Bryopsidella neglecta* (AY004766) and *Halimeda discoidea* (AB038488) were used as outgroup for the targeted Ostreobidineae family. Operational Taxonomic Units (OTUs) were defined by clustering sequences with a cut-off of 99% similarity. *Ostreobium rbc*L sequences have been deposited in Genbank under Accession Numbers MG569988 - MG570021.

### Statistical analyses

All statistical analyses were performed using the software R version 3.2.2. The mean and standard error were calculated for the following datasets: abundance and penetration depth of microborers in the host, and vertical/horizontal extension of juveniles. The non-parametric Mann and Whitney or Kruskal-Wallis tests were used as data did not meet assumptions of normal distribution (Shapiro test) and/or homoscedasticity (Bartlett test). When Kruskal-Wallis test was significant, a pairwise post-hoc analysis of Mann and Whitney was realized using Bonferroni correction^40^ and α = 0.05.

### Data availability

The *rbc*L sequences generated during and/or analysed during the current study have been deposited in Genbank under Accession Numbers MG569988 - MG570021.

## Electronic supplementary material

Supplementary Information 
