# Supplementary material for: Early skeletal colonization of the coral holobiont by the microboring Ulvophyceae Ostreobium sp
Source: Sci Rep. 2018 Feb 2;8:2293. doi: 10.1038/s41598-018-20196-5 (PMC5797222; doi:10.1038/s41598-018-20196-5)
Supplement: Supplementary file 1 — Supplementary Information [file 41598_2018_20196_MOESM1_ESM.pdf]

Early skeletal colonization of the coral holobiont by the microboring Ulvophyceae *Ostreobium* sp.

Massé A[1-2], Domart-Coulon I[1], Golubic S[3], Duché D[4], Tribollet A[2*]

[1] Sorbonne Universités - Muséum National d'Histoire Naturelle, Laboratoire MCAM UMR7245 CNRS-MNHN, 63 rue Buffon, 75005 Paris, France

[2] IRD-Sorbonne Universités (UPMC Univ, Paris 06), Laboratoire LOCEAN UMR7159 CNRS-MNHN, 4 Place Jussieu, 75005 Paris Cedex, France. E-mail : aline.tribollet@ird.fr

[3] Biological Science Center, Boston University, Boston, MA, USA

[4] Aquarium Tropical, Palais de la Porte Dorée, 293 Avenue Daumesnil, 75012 Paris, France

*corresponding author

Keywords: euendoliths, microborers, *Ostreobium* clades, coral skeleton, coral larval settlement, *Pocillopora damicornis*, colonization dynamics

SUPPLEMENTARY INFORMATION FOR MS SREP-17-34892A by Massé et al

Legend of the Supplementary Figure 1:

Phylogeny of *Ostreobium* sp. *rbc*L in *P. damicornis* adults and juveniles (Juv) (in red), their settlement substrates and seawater (SW) (in green). **a.** Maximum likelihood tree based on 578 nt *rbc*L sequence analysis: cloned OTUs (>99% similarity) were aligned with reference type strains and *Ostreobium* clones from Red Sea massive corals, with two Bryopsidales as outgroup (500 bootstraps). **b.** *Ostreobium* clades in pre-colonized substrates and in SW. **c.** Site-specific distribution of *Ostreobium* clades in *P. damicornis* type *beta* from three French aquaria, compared to those in *P. verrucosa* from Eilat Red Sea and *Pocillopora* sp. from New Caledonian reefs.

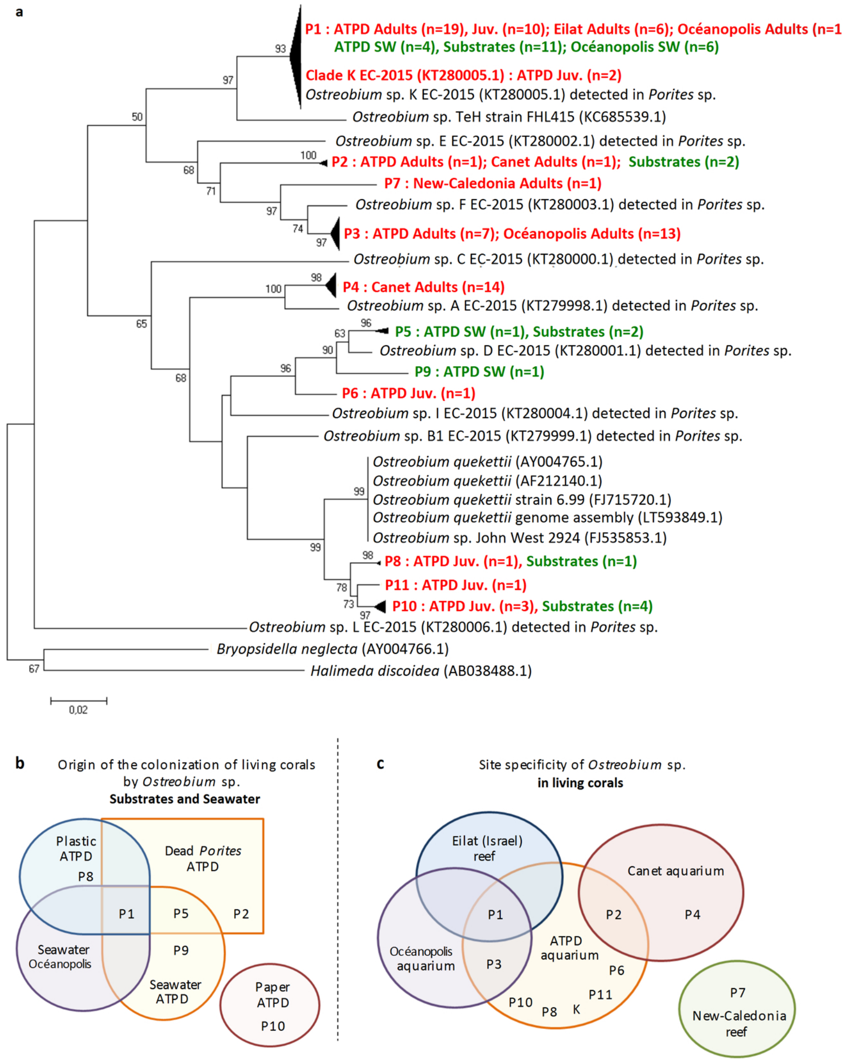
a
P1 : ATPD Adults (n=19), Juv. (n=10); Eilat Adults (n=6); Océanopolis Adults (n=1
ATPD SW (n=4), Substrates (n=11); Océanopolis SW (n=6)
Clade K EC-2015 (KT280005.1) : ATPD Juv. (n=2)
Ostreobium sp. K EC-2015 (KT280005.1) detected in Porites sp.
Ostreobium sp. TeH strain FHL415 (KC685539.1)
Ostreobium sp. E EC-2015 (KT280002.1) detected in Porites sp.
P2 : ATPD Adults (n=1); Canet Adults (n=1); Substrates (n=2)
P7 : New-Caledonia Adults (n=1)
Ostreobium sp. F EC-2015 (KT280003.1) detected in Porites sp.
P3 : ATPD Adults (n=7); Océanopolis Adults (n=13)
Ostreobium sp. C EC-2015 (KT280000.1) detected in Porites sp.
P4 : Canet Adults (n=14)
Ostreobium sp. A EC-2015 (KT279998.1) detected in Porites sp.
P5 : ATPD SW (n=1), Substrates (n=2)
Ostreobium sp. D EC-2015 (KT280001.1) detected in Porites sp.
P9 : ATPD SW (n=1)
P6 : ATPD Juv. (n=1)
Ostreobium sp. I EC-2015 (KT280004.1) detected in Porites sp.
Ostreobium sp. B1 EC-2015 (KT279999.1) detected in Porites sp.
Ostreobium quekettii (AY004765.1)
Ostreobium quekettii (AF212140.1)
Ostreobium quekettii strain 6.99 (FJ715720.1)
Ostreobium quekettii genome assembly (LT593849.1)
Ostreobium sp. John West 2924 (FJ535853.1)
P8 : ATPD Juv. (n=1), Substrates (n=1)
P11 : ATPD Juv. (n=1)
P10 : ATPD Juv. (n=3), Substrates (n=4)
Ostreobium sp. L EC-2015 (KT280006.1) detected in Porites sp.
Bryopsidella neglecta (AY004766.1)
Halimeda discoidea (AB038488.1)
93
97
50
100
68
71
97
74
97
65
98
100
96
63
90
96
68
99
99
98
78
73
97
67
0,02
b
Origin of the colonization of living corals by Ostreobium sp.
Substrates and Seawater
Plastic ATPD
P8
Dead Porites ATPD
P1
P5
P2
P9
Seawater Océanopolis
Seawater ATPD
Paper ATPD
P10
c
Site specificity of Ostreobium sp.
in living corals
Eilat (Israel) reef
Canet aquarium
P1
P2
P4
Océanopolis aquarium
ATPD aquarium
P3
P6
P11
P10
P8
K
P7
New-Caledonia reef
